# A VHH single-domain platform enabling discovery and development of monospecific antibodies and modular neutralizing bispecifics against SARS-CoV-2 variants

**DOI:** 10.1093/abt/tbae009

**Published:** 2024-05-03

**Authors:** Marisa L Yang, Tom Z Yuan, Kara Y Chan, Lin Ding, Zhen Han, Hector Franco, Carson Holliday, Shruthi Kannan, Edgar Davidson, Benjamin J Doranz, Kartik Chandran, Emily Happy Miller, Jessica A Plante, Scott C Weaver, Eunice Cho, Shweta Kailasan, Lukas Marsalek, Hoa Giang, Yasmina Abdiche, Aaron K Sato

**Affiliations:** Biopharma Department, Twist Bioscience, South San Francisco, CA 94080, United States; Biopharma Department, Twist Bioscience, South San Francisco, CA 94080, United States; Biopharma Department, Twist Bioscience, South San Francisco, CA 94080, United States; Biopharma Department, Twist Bioscience, South San Francisco, CA 94080, United States; Biopharma Department, Twist Bioscience, South San Francisco, CA 94080, United States; Biopharma Department, Twist Bioscience, South San Francisco, CA 94080, United States; Biopharma Department, Twist Bioscience, South San Francisco, CA 94080, United States; Integral Molecular, Philadelphia, PA 19104, United States; Integral Molecular, Philadelphia, PA 19104, United States; Integral Molecular, Philadelphia, PA 19104, United States; Department of Microbiology and Immunology, Albert Einstein College of Medicine, Bronx, NY 10461, United States; Department of Medicine, Albert Einstein College of Medicine, Bronx, NY 10461, United States; World Reference Center for Emerging Viruses and Arboviruses, University of Texas Medical Branch, Galveston, TX 77555, United States; Department of Microbiology and Immunology, University of Texas Medical Branch, Galveston, TX 77555, United States; World Reference Center for Emerging Viruses and Arboviruses, University of Texas Medical Branch, Galveston, TX 77555, United States; Department of Microbiology and Immunology, University of Texas Medical Branch, Galveston, TX 77555, United States; Integrated Biotherapeutics, Rockville, MD 20850, United States; Integrated Biotherapeutics, Rockville, MD 20850, United States; Eyen SE, Prague, Czech Republic; Biopharma Department, Twist Bioscience, South San Francisco, CA 94080, United States; Revelar Biotherapeutics, Inc., Bethesda, MD 20817, United States; Biopharma Department, Twist Bioscience, South San Francisco, CA 94080, United States

**Keywords:** SARS-CoV-2, omicron, VHH, neutralizing antibody, tetravalent bispecific, multivalent, synthetic library, avidity

## Abstract

Severe acute respiratory syndrome coronavirus 2 (SARS-CoV-2) continues to evolve, escape coronavirus disease 2019 therapeutics and vaccines, and jeopardize public health. To combat SARS-CoV-2 antigenic escape, we developed a rapid, high-throughput pipeline to discover monospecific VHH antibodies and iteratively develop VHH-Fc-VHH bispecifics capable of neutralizing emerging SARS-CoV-2 variants. By panning VHH single-domain phage libraries against ancestral or beta spike proteins, we discovered high-affinity VHH antibodies with unique target epitopes. Combining two VHHs into a tetravalent bispecific construct conferred broad neutralization activity against multiple variants and was more resistant to antigenic escape than the monospecific antibody alone. Following the rise of the Omicron variant, a VHH in the original bispecific construct was replaced with another VHH discovered against the Omicron BA.1 receptor binding domain; the resulting bispecific exhibited neutralization against both BA.1 and BA.5 sublineage variants. A heavy chain-only tetravalent VHH-Fc-VHH bispecific platform derived from humanized synthetic libraries held a myriad of unique advantages: (i) synthetic preconstructed libraries minimized risk of liabilities and maximized discovery speed, (ii) VHH scaffolds allowed for a modular “plug-and-play” format that could be rapidly iterated upon as variants of concern arose, (iii) natural dimerization of single VHH-Fc-VHH polypeptides allowed for straightforward bispecific production and purification methods, and (iv) multivalent approaches enhanced avidity boosting effects and neutralization potency, and conferred more robust resistance to antigenic escape than monovalent approaches against specific variants. This iterative platform of rapid VHH discovery combined with modular bispecific design holds promise for long-term viral control efforts.

## Teaser

Statement of Significance: A modular antibody platform leveraging VHH heavy-chain constructs derived from humanized synthetic libraries to rapidly generate tetravalent VHH-Fc-VHH bispecifics was developed to combat viruses like SARS-CoV-2 that exhibit rapid antigenic drift. This platform was utilized to discover new antibodies and iteratively develop neutralizing bispecifics as new SARS-CoV-2 variants arose.

## Introduction

The persistent and unpredictable evolution of severe acute respiratory syndrome coronavirus 2 (SARS-CoV-2) continues to generate new variants of concern. Each one brings with it the possibility of enhanced transmissibility and virulence as well as immune escape [1]. The alpha and delta variants caused waves of infections and millions of deaths due to their enhanced transmissibility and virulence relative to contemporaneous strains [2–4]. By contrast, the more recent Omicron variant is less virulent [5] but considerably more resistant to natural and acquired immunity, as demonstrated by vaccine breakthrough cases and the obsolescence of many emergency-use authorized (EUA) monoclonal antibodies (mAbs) [6–9]. With the possibility of more virulent variants on the horizon, expeditious pipelines for discovering novel therapeutics and updating current ones are imperative.

MAbs are a key component of the coronavirus disease 2019 (COVID-19) response, complementing existing SARS-CoV-2 antivirals. MAbs are efficacious, can have minimal drug interactions, and can be delivered in a single intravenous injection [10]. Unlike vaccines, mAbs do not require a functioning immune system to be effective, providing immunocompromised individuals with a therapeutic option. Despite these benefits, a single-point mutation in the SARS-CoV-2 spike (S) protein can render a mAb ineffective [11]. The SARS-CoV-2 S can accommodate a surprising number of mutations while maintaining high affinity for the angiotensin-converting enzyme 2 (ACE2) receptor [12, 13]. SARS-CoV-2 has even escaped antibodies targeting relatively conserved epitopes, including S309 [14], a class 3 antibody that targets a conserved epitope that includes the N343 glycan, and ADG-20 [15], a class 4 antibody that binds the conserved CR3022 site [16].

Two strategies that have been proposed to suppress antigenic escape include mAb cocktails and bispecifics. Although both can minimize escape, bispecifics could potentially do so with fewer molecules (one versus multiple), making them easier to manufacture, formulate, and administer in low doses than mAb cocktails. Further, joining antibodies in a multivalent construct could boost avidity to the SARS-CoV-2 virus, which harbors multiple copies of the S trimer protein, thus strengthening neutralizing activity [17, 18]. Plug-and-play bispecifics can be built with single-domain (VHH) antibodies to enable rapid development and iteration. Numerous anti-SARS-CoV-2 VHHs have been and continue to be discovered [18–27], providing a rich toolbox of modular parts for bispecific development. Yet, this toolbox remains critically underutilized in the COVID-19 response. Here, we describe our pipeline, which provides a streamlined approach to single-domain antibody discovery and bispecific iteration in order to rapidly address emerging SARS-CoV-2 variants.

## Results

**Figure 1:**
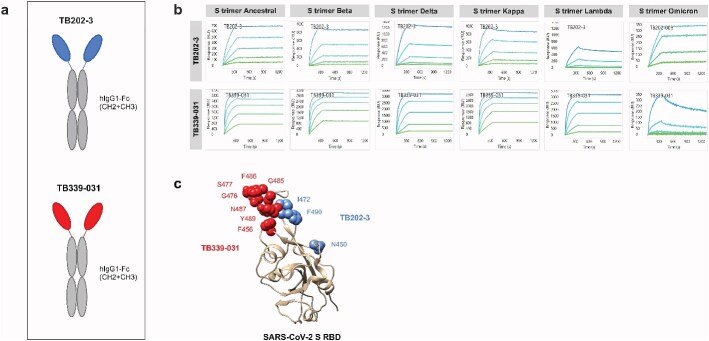
TB202-3 and TB339-031 antibody candidates exhibit high-affinity binding profiles and complementary epitopes on SARS-CoV-2 S RBD. (A) Diagrams of TB202-3 and TB339-031 VHH-Fc antibodies. Blue and red represent the variable heavy region of TB202-3 and TB339-031, respectively. Gray represents the Fc constant region (CH2 + CH3). (B) TB202-3 and TB339-031 VHH-Fc antibodies demonstrate binding to multiple SARS-CoV-2 S trimer variants. Binding profiles were determined by SPR in an avidity boosted assay format and as such, did not undergo fitting. (C) Epitope mapping of TB339-031 and TB202-3 using alanine scanning shotgun mutagenesis approach on Ancestral SARS-CoV-2 S RBD (PDB 6LZG) (beige ribbon). F456, G476, S477, G485, F486, N487, and Y489 were identified as essential for the interaction between TB339-031 and the ancestral RBD. Critical residues for the interaction between TB202-3 and the ancestral RBD were previously identified [27].

We previously described the discovery of dozens of neutralizing VHHs from synthetic phage libraries panned against the ancestral SARS-CoV-2 S1 monomer protein (GenBank QHD43416.1, residues 16–685) [27]. These libraries were built for developability—manufacturing liabilities were removed (such as some sequences linked to post-translational modifications, cryptic splice sites, and commonly used nucleotide restriction sites). From these libraries, we identified a VHH-Fc antibody—TB202-3 (Fig. 1a)—that binds the ancestral S trimer with picomolar potency, neutralizes the authentic ancestral SARS-CoV-2 *in vitro* and *in vivo*, and neutralizes SARS-CoV-2 beta pseudovirus *in vitro* [27]. In pseudovirus assays conducted independently as part of the Coronavirus Immunotherapeutic Consortium (CoVIC), TB202-3 (CoVIC-094) neutralized the alpha, beta, and gamma variants but not Delta and Epsilon [28]. We evaluated the binding specificity of TB202-3 with a panel of variant S trimers using surface plasmon resonance (SPR) in an avidity-boosted assay format. We observed that TB202-3 bound tightly to all variants tested, albeit less so to Lambda S trimer (Fig. 1b). These data point to L452Q or F490S, which are present in Lambda variants, as a vulnerability of TB202-3. Per our previous study [27], alanine scanning revealed residues N450, I472, and F490 as essential for the interaction between TB202-3 and ancestral S receptor binding domain (RBD) (Fig. 1c). The relatively weak association between TB202-3 and the Lambda S trimer may be explained by the L452Q and F490S mutations in the Lambda S protein, as these residues may be critical binding sites of TB202-3 (Supplementary Table 1).

In parallel, we leveraged our high-throughput pipeline [27] to discover candidate VHHs that would complement TB202-3 in a bispecific construct. Using the same synthetic VHH phage libraries we used in our previous campaign [27], we executed an immobilized protein-based biopanning strategy against the Beta S1 monomer protein, as the Beta variant was a widely circulating variant at the time. After four rounds of panning, we performed an enzyme-linked immunosorbent assay (ELISA) against the Beta S1 protein to assess the binding of individual phage clones. Clones that elicited a signal that was at least 3-fold higher than the target protein compared to the bovine serum albumin (BSA) control protein were sequenced, resulting in 31 initial lead clones with unique sequences. For further characterization and development, these 31 candidates were reformatted into a VHH-Fc fusion containing the Fc region of human IgG1 for further characterization and development.

We characterized the binding affinity of our antibody candidates using SPR in an avidity-boosted assay format and showed that lead candidate TB339-031 (Fig. 1a) bound tightly to all variant S trimers tested except the Omicron, which showed weaker binding (Fig. 1b). We performed epitope mapping via a shotgun mutagenesis approach (alanine scanning) to clarify which residues of TB339-031 may be responsible for its weaker binding to the Omicron RBD. Epitope mapping was performed using SARS-CoV-2 ancestral RBD to facilitate a comparison with our previous antibody candidate TB202-3. We identified F456, G476, S477, G485, F486, N487, and Y489 as critical epitope contacts on the ancestral RBD used in binding to TB339-031 (Fig. 1c, Supplementary Fig. 1). These data suggest that S477 represents a putative vulnerability for TB339-031 and may explain its weak binding to Omicron, which contains the S477N mutation. By comparing the critical contacts deduced for both TB339-031 and TB202-3 via shotgun mutagenesis, we concluded that the two antibodies target closely adjacent epitopes (Fig. 1c).

**Figure 2:**
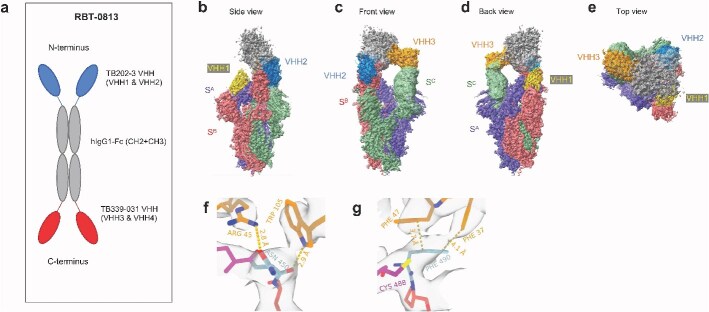
TB202-3 and TB339-031 were combined into RBT-0813, a VHH-Fc-VHH bispecific. Cryo-EM reveals a three-point interaction between RBT-0813 and the SARS-CoV-2 Ancestral S trimer. Purple, red, and green represent monomers of the spike trimer, further denoted as chain A (S^A^), chain B (S^B^), and chain C (S^C^), respectively. VHH1 and VHH2 correspond to the N-terminal TB202-3 arms. VHH3 corresponds with one of the C-terminal TB339-031 arms, VHH4 could not be determined due to low resolution. VHH1 is depicted in gold, VHH2 in blue, VHH3 in orange. Constant fragment is represented in gray. (A) Diagram of RBT-0813, a VHH-Fc-VHH bispecific composed of TB202-3 and TB339-031. Blue and red represent the variable heavy region of TB202-3 and TB339-031, respectively. Gray represents the Fc constant region (CH2 + CH3). (B) Side view: VHH1 (gold) is bound to the RBD down domain on spike (S^A^). VHH2 (blue) is bound to the neighboring RBD up domain on spike (S^B^). (C) Front view: VHH2 (blue) is bound to one of the RBD up domains (S^B^), while VHH3 (orange) is bound flexibly to the second RBD up domain (S^C^) and they both share a strong constant fragment density. (D) Back view: VHH3 (orange) is bound flexibly to the second RBD up domain (S^C^), while VHH1 (gold) is bound to the RBD down domain (S^A^). (E) Top view: VHH1 (gold) is located at the bottom, VHH2 (blue) in the upper right corner, both partially covered by the constant fragment, VHH3 (orange) is on the left. (F) ARG 45, TRP 105 (both from VHH) are interacting with sidechain (ARG 45) and backbone (TRP 105) of ASN 450 (spike). These finding are in agreement with results from the mutagenesis study, and benefits the importance of ARG 45: ASN 450 interaction in integrity of spike: Ab complex. (G) The possible stacking interactions between PHE 490 (spike) and PHE 37, 47 (both from VHH) were suggested during manual model inspection and are in agreement with results from the mutagenesis study.

Due to the complementary binding specificities of TB202-3 and TB339-031, we combined them into a bispecific, tetravalent, and symmetric VHH-Fc-VHH construct (known as RBT-0813) (Fig. 2a). Our rationale for this design was three-fold: (i) the Fc domain could confer increased serum half-life, normal Fc effector functionality, conform to standard platform purification processes (protein A chromatography), and allow for natural homodimerization of a single polypeptide chain into a multivalent final product; (ii) tetravalency would afford flexibility and avidity-boosting effects in enhancing binding to the S trimer; and (iii) bispecificity would broaden the epitope footprint to minimize antigenic escape [21].

Next, we analyzed the structure of RBT-0813 bound to the ancestral S trimer using cryo-electron microscopy (cryo-EM). Structures derived from PDB:6x2b and AlphaFold2 predictions were used to rigid-body fit the map densities of the spike protein and VHH domains of RBT-0813, respectively, followed by subsequent manual and molecular dynamics modeling to build more precise models. The structure reconstruction revealed densities for 3 out of the 4 VHHs present on RBT-0813, as well as a density corresponding to the Fc domain (Fig. 2b–e) that aided in confirming the positional identity of the Fc-tethered VHH moieties, namely TB202-03 (N-terminal, named herein as VHH1 and VHH2) or TB339-031 (C-terminal, named herein as VHH3). The location or presence of the fourth VHH (from the TB339-031 parent, named herein as VHH4) could not be confirmed. Two VHH densities (resolved at 3.2–3.3 Å) corresponded to the N-terminal TB202-3 arms, one bound to a “down” RBD on one spike (S^A^) and another bound to an “up” RBD on another (S^B^). The two TB202-3 arms (VHH1 and VHH2) bind highly similar but not identical epitopes on the S trimer. One epitope is assigned to the RBD of S^B^, and the other spans the RBD of S^A^ and the N-terminal domain (NTD) of the neighboring S^B^ (Supplementary Table 1 summarizes putative interacting residues in each epitope). This minor difference between the two TB202-3 epitopes indicates flexibility in the binding of at least the TB202-3 arms to the S trimer and also demonstrates that multiple VHH arms of the RBT-0813 construct can bind to the spike trimer simultaneously through the binding of largely similar yet slightly different epitopes on the spike trimer—thus allowing the construct the opportunity to engage in avidity-boosting effects. From the previous alanine scanning mutagenesis experiment, we identified residues N450, I472, and F490 as critical in TB202-3. Similarly, Cryo-EM confirmed the presence of residues N450, I472, and F490 in the TB202-3 epitope, identified either by close proximity or by a combination of manual and computational strategies, although I472 was only confirmed in the TB202-3 epitope on S^B^ (Fig. 2f and g, Supplementary Table 1). We deduced the third VHH density, bound to the second “up” RBD (S^C^), to be one of the C-terminal TB339-031 arms (termed VHH3) based on its connection to the Fc domain, the stoichiometry of RBT-0813, and the expected binding site of TB339-031 from the alanine scan. The epitope of this putative TB339-031 density could not be determined in detail due to the low resolution of the density (around 6 Å) in that area of the cryo-EM map.

**Figure 3:**
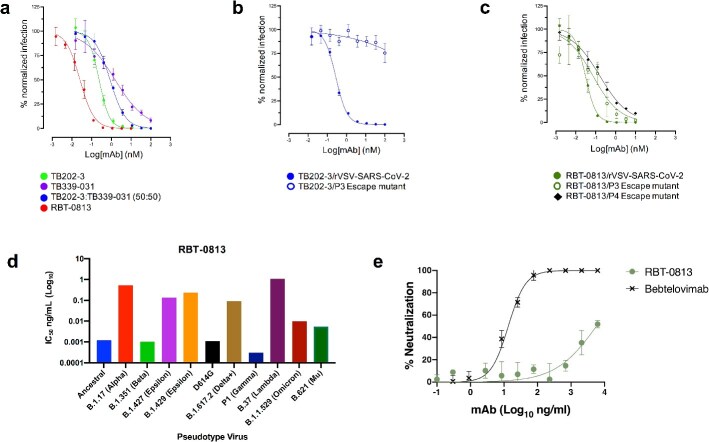
Bispecific exhibits increased resistance to antigenic escape and broad neutralization of many SARS-CoV-2 variants, but with limited effect against Omicron BA.5 in authentic virus neutralization assays. (A) Pseudovirus neutralization assay against rVSV-SARS-CoV-2 (Wuhan 1) with TB202-3, TB339-031, TB202-3 + TB339-031 cocktail, and RBT-0813 bispecific. Symbols represent the sample mean, error bars represent the standard deviation, and curves were fit by a nonlinear fit log(inhibitor) versus normalized response—variable slope function in Prism. Data are representative of a single experiment performed with independent triplicates. (B) Antigenic escape of rVSV-SARS-CoV-2 from TB202-3 is noted after three passages. (C) Incomplete antigenic escape of rVSV-SARS-CoV-2 from RBT-0813 is noted after four passages. (D) In pseudovirus assays, RBT-0813 exhibits varied levels of neutralization of SARS-CoV-2 variants (IC_50_ < 0.52 ng/ml), with limited effect particularly against Lambda (IC_50_ = 1.077 ng/ml). (E) Authentic virus neutralization assay against SARS-CoV-2 Omicron BA.5. Symbols represent the sample mean, error bars represent the standard deviation, and curves were fit by the log(agonist) versus response function of Prism. Data are representative of a single experiment performed with independent triplicates.

To analyze antigenic escape, we generated escape mutants by serial passage of a replication-competent vesicular stomatitis virus (VSV) bearing the ancestral SARS-CoV-2 spike (rVSV-SARS-CoV-2 Wuhan) pseudovirus under antibody pressure. We selected escape mutants against parental clones TB202-3 and TB339-031 (both in a monospecific bivalent VHH-Fc format) and the bispecific RBT-0813. rVSV-SARS-CoV-2 Wuhan contains W64R, A372T, and H655Y mutations, which arose during viral rescue and are previously described [29, 30]. TB339-031 did not elicit any escape mutants because it was a relatively poor neutralizer of the ancestral spike (purple curve in Fig. 3a). Escape mutants were elicited within three passages in the presence of TB202-3. In contrast, three passages in the presence of RBT-0813 resulted in only minimal changes to neutralization. A fourth passage did elicit escape somewhat but still maintained substantial neutralization, indicating that the bispecific RBT-0813 is more resistant to antigenic escape than the monospecific TB202-3 (Fig. 3b and c). We identified F490S, Y655H, R685G, and F759S in a TB202-3 escape clone and F275L, N354D, Y655H, R685G, and F759S in two RBT-0813 escape clones. Y655H represents a reversion of the H655Y mutation present in the parental rVSV-SARS-CoV-2. R685G and F759S have been observed previously [29, 30] and may represent adaptations to growth in tissue culture. The sole RBD mutation in the TB202-3 escape clone (F490S) corroborates the TB202-3 epitope defined by alanine scanning and is consistent with our cryo-EM structure (Figs 1c and 2g). In the RBT-0813 escape clone, RBD mutations F275L and N354D were detected in addition to the potentially unrelated Y655H, R685G, and F759S mutations.

To evaluate the resistance of RBT-0813 to real-world antigenic escape, we assayed the neutralizing effects of RBT-0813 against SARS-CoV-2 Ancestral, B.1.17 Alpha, B.1.351 Beta, B.1.427 Epsilon, B.1.429 Epsilon, D614G, B.1.617.2 Delta+, P1 Gamma, B.37 Lambda, B.1.1.529 Omicron, and B.621 Mu (Fig. 3d) in pseudovirus assays, using VSV pseudotyped with SARS-CoV-2 S. RBT-0813 was broadly neutralizing but less potent against the Lambda (IC_50_ = 1.077 ng/ml) variant (Fig. 3d), which possesses two mutations (L452Q and F490S) in the TB202-3 epitope. During the preparation of this manuscript, Omicron subvariant BA.5 became the dominant strain in the United States [31]. We therefore tested RBT-0813 in an authentic virus assay against BA.5 (Fig. 3e). RBT-0813 was poorly neutralizing against this variant, potentially due to a “double knock out” of critical contacts for both specificities—mutation L452R impacting the TB202-3 epitope and mutations S477N and F486V impacting the TB339-031 epitope.

**Figure 4:**
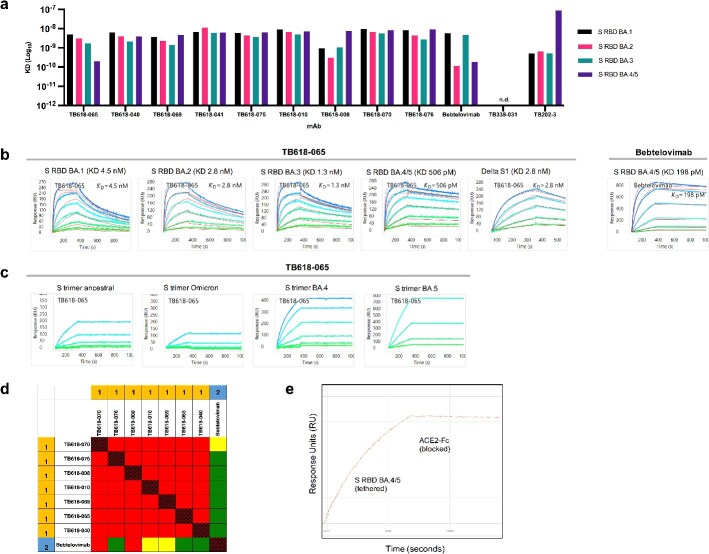
TB618-065 exhibits high-affinity binding profiles to SARS-CoV-2 Omicron variants, and unique epitope. (A) Apparent K_D_ (M) binding affinity of VHH-Fc antibodies, demonstrating binding to multiple SARS-CoV-2 variant S RBDs in the picomolar to nanomolar range. “N.d.” signifies “not detectable.” Binding profiles were determined by SPR. (B) SPR kinetics sensorgrams of TB618-065 against various SARS-CoV-2 variants, and bebtelovimab against Omicron S RBD BA.4/5. TB618-065 binds to Omicron S RBD BA.1, BA.2, BA.3, BA.4/5, and Delta S1 with picomolar to single-digit nanomolar affinity. (C) SPR kinetics sensorgrams of TB618-065 to various S trimer subvariants in an avidity-boosted assay format. (D**)** SPR epitope competition binning assay of SARS-CoV-2 S RBD Omicron binding antibodies. Antibodies that exhibited high affinity from kinetics assay were matrixed in a cross-competition SPR assay. Red, yellow, and green cells in the heat map represent competitive, weakly competitive, and non-competitive blocked analyte and ligand pairs, respectively. Antibodies that did not exhibit self-blocking behavior were omitted from the analysis. All TB618 leads tested formed a distinct bin from EUA mAb Bebtelovimab. (E) TB618-065 exhibits blocking effects of ACE2-Fc in a SPR competition assay. TB618-065 was coupled to the biosensor surface, then sequentially injected with SARS-CoV-2 Omicron RBD BA.4/5, then subsequently ACE2-Fc.

Given the escape of BA.5 from RBT-0813, we revisited our rapid discovery pipeline to identify VHHs that neutralize Omicron subvariants with the goal of updating the VHH modules that comprised our bispecific construct. We panned the same two VHH phage libraries, we used to discover TB202-3 and TB339-031 against the Omicron BA.1 S RBD, since BA.1 was the first dominant Omicron variant and was a widely circulating strain at the time. Hits identified by phage ELISA screening were reformatted to VHH-Fc and characterized by their binding to ancestral S trimers, Omicron subvariant S trimers, and Omicron subvariant RBDs using high-throughput SPR. Multiple candidates—TB618-065, -040, -069, -041, -075, -010, -008, -070, -076, and emergency use authorization (EUA) antibody bebtelovimab—bound with apparent monovalent affinities spanning pico- to nanomolar affinity to the Omicron subvariant S RBDs (Fig. 4a). Notably, TB618-065 exhibited apparent monovalent affinities of pico- to single-digit nanomolar to the Omicron subvariant S RBDs and Delta S1, as well as avidity-boosted binding to S trimer variants (Fig. 4b and c). As a benchmark comparison, bebtelovimab and TB618-065 exhibited a monovalent affinity for Omicron S RBD BA.4/5 of 198 and 506 pM, respectively (Fig. 4b). TB202-3 maintained binding to all tested subvariants of Omicron RBD (BA.1, BA.2, BA.3, and BA.4/5), albeit with a lower affinity for BA.4/5 (Fig. 4a). In contrast, TB339-031 completely lost binding to all tested subvariants of Omicron RBD (Fig. 4a)—supporting the need for new VHHs that target the Omicron variant to replace the TB339-031 arm of the RBT-0813 bispecific.

**Figure 5:**
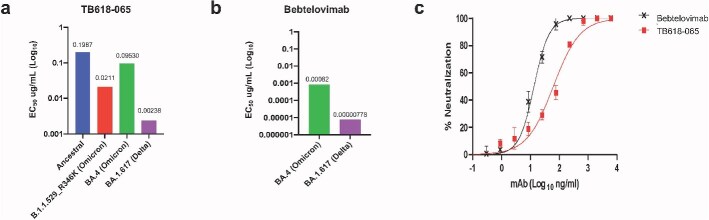
TB618-065 exhibits broad neutralization of SARS-CoV-2 Omicron in pseudovirus and authentic virus assays. (A) Pseudovirus neutralization assay shows TB618-065 to exhibit broad neutralization (EC_50_ < 199 ng/ml) of SARS-CoV-2 ancestral, Omicron (BA.1.1.529_R346K), Omicron BA.4, and Delta. (B) Pseudovirus neutralization assay shows EUA antibody Bebtelovimab to exhibit broad neutralization (EC_50_ < 0.82 ng/ml, EC_50_ < 0.0078 ng/ml) of SARS-CoV-2 Omicron BA.4 and Delta BA.1.617, respectively. Bebtelovimab was not tested against Ancestral and B.1.1.529_R346K. (C) Authentic virus neutralization assay against SARS-CoV-2 Omicron BA.5 with TB618-065 and Bebtelovimab. Symbols represent the sample mean, error bars represent the standard deviation, and curves were fit by the log(agonist) versus response function of Prism. Data is representative of a single experiment performed with independent triplicates.

We next assayed the cross-competition of these top candidates and the EUA antibody bebtelovimab with the BA.4/BA.5 RBD using HT-SPR (Fig. 4d). All TB618 leads that were tested binned together in the same competition bin 1, whereas bebtelovimab formed a distinct bin of its own. In SPR assays, TB618-065 blocked the interaction between ACE2 and BA.4/5 RBD (Fig. 4e). Because TB618-065 bound BA.4/5 RBD most potently of the TB618 leads and showed no cross-competition with bebtelovimab (Fig. 4d), we compared it alongside bebtelovimab in pseudovirus and authentic virus neutralization assays (Fig. 5a–c). TB618-065 potently neutralized Ancestral, Omicron (B.1.1.529_R346K), Omicron BA.4, and Delta BA.1.617 pseudoviruses (EC_50_ < 199 ng/ml) (Fig. 5a). Bebtelovimab was a marginally better neutralizer of Delta and Omicron BA.4 pseudoviruses (Fig. 5b) as well as authentic BA.5 (Fig. 5c).

**Figure 6:**
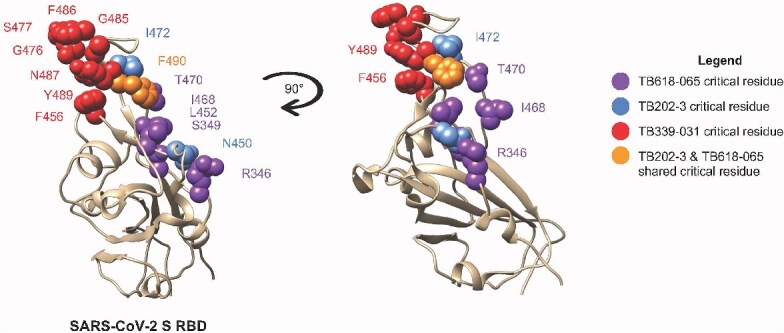
Alanine scanning mutagenesis epitope mapping reveals critical epitopes of TB618-065 to SARS-CoV-2 Ancestral RBD (beige ribbon). Epitope mapping of TB618-065, TB339-031, and TB202-3 onto Ancestral SARS-CoV-2 S RBD (PDB 6LZG), using alanine scanning shotgun mutagenesis approach. Overlay plots shown in different views (front view, 90° rotated view) reveal the unique critical epitope contacts for TB618-065 (purple), TB202-3 (blue), TB339-031 (red), and shared critical contact (F490) for TB202-3 and TB618-065 (orange). R346, S349, L452, I468, T470, and F490 were identified as essential for the interaction between TB618-065 and the ancestral RBD. Critical residues for the interaction between TB202-3 and TB339-031 were identified through a previous epitope mapping alanine scanning mutagenesis experiment (Fig. 1c, 27).

**Figure 7:**
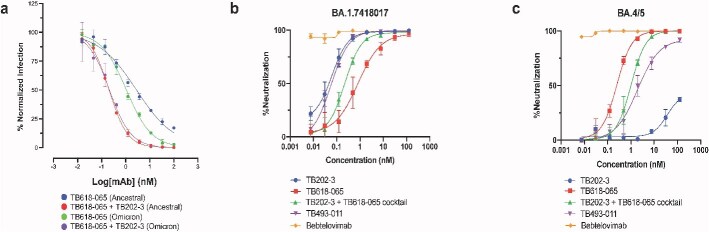
(A) TB202-3 + TB618–065 cocktail exhibit neutralization against rVSV-SARS-CoV-2 (Wuhan) or rVSV-SARS-CoV-2 (Omicron BA.1). Symbols represent the sample mean, error bars represent the standard deviation, and curves were fit by a nonlinear fit log(inhibitor) versus normalized response—variable slope function in Prism. Data are representative of a single experiment performed with independent triplicates. (B-C) TB202-3, TB618-065, TB202-3 + TB618-065 cocktail, TB493-011, and bebtelovimab exhibit varying levels of neutralization against rVSV-SARS-CoV-2 (Omicron BA.1.7418017) and rVSV-SARS-CoV-2 (Omicron BA.4/5), respectively.

To map the epitope of TB618-065, we performed alanine scanning mutagenesis on ancestral RBD, identifying R346, S349, L452, I468, T470, and F490 as critical residues (Fig. 6, Supplementary Fig. 2). Combined with the neutralization data, these data show that TB618-065 is a broad-spectrum SARS-CoV-2 neutralizer that targets a distinct epitope from bebtelovimab. Preliminary neutralization experiments demonstrated that the combination of TB202-3 and TB618-065 (in cocktail format) improved neutralizing activity against ancestral and Omicron BA.1 rVSVs relative to the TB618-065 monospecific VHH-Fc construct (Fig. 7a). Thus, we combined TB202-3 and TB618-065 into a tetravalent bispecific construct, effectively replacing the TB339-031 arm of RBT-0813 with TB618-065 to generate the bispecific TB493-011. We observed TB493-011 exhibited stronger binding in an ELISA against recombinant SARS-CoV-2 RBD BA.1 and BA.4/5 compared to the binding behavior of TB202-3 or TB618-065 monospecific antibodies (Supplementary Fig. 3a and b). We then tested the TB493-011 bispecific alongside the TB202-3 and TB618-065 monospecifics alone as well as TB202-3 + TB618-065 cocktail in a pseudovirus experiment against an available Omicron BA.1 sublineage (BA.1.7418017) rVSV and BA.4/5 rVSV. Neutralization results showed similar but not identical trends to the ELISA binding data—indicating that ELISA binding was not fully representative of neutralization activity. TB493-011 showed improved neutralization activity (NT_50_ 0.05 nM) against BA.1.7418017 relative to TB618-065 alone (NT_50_ 0.81 nM) and TB202-3 + TB618-065 cocktail (NT_50_ 0.22 nM); TB493-011 showed similar neutralization activity as TB202-3 (NT_50_ 0.06 nM) (Fig. 7b). However, while TB202-3 showed strong neutralization activity against Omicron BA.1.7418017, its neutralization activity dropped significantly against Omicron BA.4/5. Although TB202-3 exhibited reduced neutralization (NT_50_ 36.41 nM) against the BA.4/5 subvariant, TB493-011 and TB202-3 + TB618-065 cocktails still maintained strong neutralization (NT_50_ 1.92 nM, NT_50_ 1.10 nM), likely relying on the strong neutralizing activity of TB618-065 (NT_50_ 0.24 nM) (Fig. 7c). Bebtelovimab exhibited strong neutralization against both variants (NT_50_ < 0.0007 nM, NT_50_ < 0.0007 nM) (Fig. 7b and c). The findings of this pseudovirus experiment illustrate how the TB493-011 bispecific exhibits neutralization against multiple subvariants, and can still demonstrate neutralization even when one of its “modules” like TB202-3 loses some activity. As such, the bispecific TB493-011 exhibits promising qualities compared to its constituent monospecific antibodies when faced with multiple variants.

## Discussion

The Omicron variant represented a major antigenic shift in the evolution of a virus that had previously been characterized by smaller antigenic drifts [13]. This step-change antigenic event has made many therapeutic antibodies obsolete, with only cilgavimab, bebtelovimab, and imdevimab retaining neutralizing activity against BA.4 and BA.5 and only bebtelovimab against all Omicron subvariants at the time [6, 32–35]. RBT-0813, TB618-065, and TB493-011 joined the minority of antibodies still active against Omicron subvariants. Nevertheless, the dismal fate of most therapeutic SARS-CoV-2 antibodies—even those targeting relatively conserved epitopes—suggests that antibodies still retaining activity may soon become obsolete, especially if they are used in the clinic.

The three lead VHH sequences described in this paper (TB202-3, TB339-031, and TB618-065) were derived from two synthetic libraries of >10^9^–10^10^ size: TB339-031 and TB618-065 were discovered from a humanized llama nanobody library with shuffled, llama-based CDR diversity. TB202-3 was discovered from a humanized llama nanobody library constructed using natural llama CDR1/2 sequences and human CDR3s identified from human naïve and memory B cells. Both libraries were constructed from solid-phase-synthesized oligonucleotides via a silicon-based nanowell platform [36]. In addition to enabling precise sequence diversification, this synthetic approach allowed us to remove potential manufacturing liabilities and other problematic sequences, reducing the likelihood that such sequences will impede development and manufacturing. These synthetic libraries are valuable sources of high-affinity, neutralizing antibodies against past, current, and potentially future SARS-CoV-2 variants of concern.

RBT-0813 demonstrated the potential of the tetravalent bispecific format—yielding increased neutralization potency compared to its constituent monospecifics and also the cocktail, as well as increased resistance to escape. Yet, although bispecifics present more diversity in binding sites, the escape of Omicron BA.5 from RBT-0813 demonstrated the construct’s lingering vulnerabilities. However, our discovery pipeline enabled us to rapidly generate a potent neutralizer of BA.5 (TB618-065) with activity that rivals bebtelovimab in authentic viruses. We then demonstrated further how our platform promotes rapid iteration, as exemplified by the modification of RBT-0813 to replace the TB339-031 VHH arm with TB618-065. TB493-011’s neutralization of both BA.1.7418017 and BA.4/5 rVSV illustrates the utility of a bispecific format that possesses an enlarged epitope footprint compared to its monospecific parentals.

Additionally, TB493-011 exhibited increased potency compared to the cocktail against BA.1.7418017—potentially due to additional binding sites available per molecule in the TB493-011 tetravalent bispecific format compared to a monospecific VHH-Fc format. However, against BA.4/5 rVSV, we recognized that TB493-011 showed less activity compared to the cocktail. We may postulate that the orientation of TB493-011 may contribute to a difference in activity against certain variants. In this bispecific construct, the TB202-3 and TB618-065 heavy domains sit on the N-terminal end and C-terminal end, respectively—whereas in its monospecific VHH-Fc format, the TB618-065 heavy domain sits on the N-terminal end. It has been shown previously that the orientation of the parental modules that comprise a bispecific can impact neutralization efficacy—against certain variants, it may result in a synergistic effect compared to cocktail format, but against others, it may not [37]. With this in mind, future work may involve additional iterations and modulations on the bispecific components as new variants arise. It is imperative to consider each VHH module’s critical residues and vulnerabilities, as well as the variants’ relevant mutations in developing further bispecific constructs that can be fine-tuned against a rapidly changing virus.

With the rapid evolution of SARS-CoV-2, it may be inevitable that most, if not all, antibody therapeutics will eventually escape. In this study, multiple experiments utilized bebtelovimab as a benchmark antibody, as it was an effective EUA mAb at the time. However, over time, bebtelovimab has eventually been escaped and since removed from EUA approval [38]. Thus, it is advantageous to be equipped with a ready-to-use and modular platform in the race against evolving variants. The platform we have utilized in this antibody discovery campaign is well equipped for this; it leverages a humanized library, a heavy chain-only antibody format, and a high-throughput approach to antibody discovery and characterization. The highly neutralizing VHH antibodies in this manuscript were discovered directly from a naïve humanized library, which lends itself to faster discovery of effective candidates. Humanized libraries and Fc-bound constructs may allow for reduced immunogenicity, human effector functions, and increased serum half-life in humans [39–41]. A heavy chain-only antibody format allows for an extremely modular, plug-and-play format to invite use of various bispecifics or multispecifics in various formats; in a tetravalent VHH-Fc-VHH bispecific format, the single polypeptide allows for simplicity in production and purification (Supplementary Fig. 4), as it can undergo common purification techniques and also self-dimerizes into the resulting tetravalent format—thus, it may also lend itself to straightforward application in mRNA delivery formats, thereby bypassing GMP protein production and expediting the drug discovery to patient timeline. Finally, the high-throughput approach to antibody synthesis, production, and characterization allows for rapid validation and iteration. Given the unpredictability of the COVID-19 pandemic, the combination of a high-throughput VHH discovery pipeline and a plug-and-play bispecific design format can be deployed as a rapid-response platform to address emerging SARS-CoV-2 variants.

## Materials and methods

### Phage library generation

Synthetic phage libraries were generated, panned, and screened as described previously [27]. Two fully synthetic phage libraries were designed for VHH discovery: a humanized llama nanobody library with shuffled, llama-based CDR diversity and a humanized llama nanobody library constructed using natural llama CDR1/2 sequences and human CDR3s identified from human naïve and memory B cells. For both libraries, CDRs were screened to remove manufacturing liabilities (such as sequences linked to post-translational modifications), cryptic splice sites, and commonly used nucleotide restriction sites; synthesized as oligo pools (Twist Bioscience); and inserted into a partially humanized DP-47 framework [42]. The llama FW2 region was included to maintain stable expression as a heavy-chain only antibody. VHH cassettes were finally cloned into the pADL-22c phagemid display vector (Antibody Design Labs) via SfiI and electroporated into TG1 *Escherichia coli* cells (Lucigen) to generate the phage libraries. Each purified phage library had an estimated 10^9^–10^10^ diversity as determined by the dilution series of colony forming units per milliliter in 2YT agar plates containing 100 μg/ml carbenicillin.

### Phage panning, screening, and sanger sequencing

Phage particles were blocked with phosphate-buffered saline (PBS) with 0.5% BSA. For immobilized protein panning, a 96-well Maxisorp plate (Thermo) was blocked with PBS/0.5% Tween +0.5% BSA; blocked phage particles were then depleted for nonspecific binders on this “depletion plate.” Additionally, recombinant SARS-CoV-2 Beta S1 protein was passively adsorbed on a 96-well Nunc Maxisorp plate using sodium carbonate pH 9.5, named herein as the “selection plate.” Selection plate was then blocked with PBS/0.5% Tween +0.5% BSA. Phage supernatant depleted of nonspecific binders were transferred to selection plate and allowed to bind for 1 hr at room temperature (RT) to select for binders with gentle nutation. Following incubation, the selection plate was washed several times with PBS/0.5% Tween to remove non-binding clones. For biotinylated bead-based panning, SARS-CoV-2 Omicron RBD (Acro SPD-C82E4) was mixed with M-280 beads, then washed with PBS/0.5% Tween to remove unbound protein and used as a panning target for four rounds of panning. Phage supernatant depleted of nonspecific binders on unbound M-280 streptavidin beads (Thermo) was then transferred to the bead mixture containing bound biotinylated SARS-CoV-2 Omicron RBD and allowed to bind for 1 hr at RT to select for binders with gentle nutation. Following incubation, beads were washed several times with PBS/0.5% Tween to remove non-binding clones. Remaining bound phages from either panning method were eluted with trypsin for 30 min at 37°C. The output supernatant enriched in binding clones was amplified in TG1 *E. coli* cells and rescued with M13K07 helper phage (Antibody Design Labs) to use as an input phage for the next round of selection, with each round increasing the wash cycles and lowering the total amount of antigen present.

Bacterial colonies containing the phagemid display vector were isolated on 2YT agar plates with 100 μg/ml carbenicillin, and single colonies were picked using QPix 420 (Molecular Devices) into 384-well plates containing 2YT with M13KO7 helper phage to express phage for use in ELISAs. Phage ELISAs were conducted using Nunc 384-well Maxisorp plates (Thermo) with passively absorbed SARS-CoV-2 Beta S1 protein or SARS-CoV-2 S RBD Omicron. Anti-M13 antibody conjugated to horseradish peroxidase (HRP) (Sino Biological 11973-MM05T-H) was used to detect the presence of bound phages following the addition of 3,3′,5,5′-tetramethylbenzidine (TMB) substrate. Clones that demonstrated three-fold binding over BSA background were submitted for rolling circle amplification (RCA) and Sanger sequencing to GENEWIZ using phiS4 (GCGGATAACAATTTGAATTCAAGGAGACAG) primer to identify the VH.

### Reformatting, expression, and purification of monoclonal antibodies

VHH single-domains of anti-SARS-CoV-2 S1 antibodies were reformatted to VHH-Fc or VHH-Fc-VHH for DNA back-translation, synthesis, and cloning into the mammalian expression vector pTwist CMV BG WPRE Neo utilizing the Twist Bioscience eCommerce portal. Clonal genes were delivered as purified plasmid DNA ready for transient transfection in HEK Expi293 cells (Thermo Fisher Scientific). Cultures in a volume of 1 ml were grown for 4 days, harvested, and purified using Protein A resin (PhyNexus) on the Hamilton Microlab STAR platform into 42 mM citrate and 167 mM HEPES, pH 6.0. CE-SDS was used to determine antibody purity and confirm molecular weight. A subset of antibodies selected for pseudovirus and live virus assays were expressed in 30 ml cultures using the same expression system. Cultures were grown for 4 days, harvested, and purified with Phynexus Protein A resin tips on the Hamilton Microlab STAR automated liquid-handling systems. Purified antibodies were concentrated using Amicon, 30 kDa cutoff spin filters. All antibodies were eluted with 50 mM sodium citrate, followed by 1 M HEPES neutralization buffer to ~pH 6.5.

### SPR affinity measurements, epitope binning, and ACE2 blocking assays of anti-SARS-CoV-2 antibodies

SPR kinetics experiments were performed on a Carterra LSA SPR biosensor equipped with a HC30M chip (Carterra) at 25°C in HBS-TE (10 mM HEPES pH 7.4, 150 mM NaCl, 3 mM EDTA, 0.05% Tween-20). Antibodies were amine-coupled to the sensor chip by EDC/NHS activation, followed by quenching with ethanolamine HCl pH 8.0. A 7-point injection series ranging from 0 to 33 nM or 0 to 100 nM of antigen were flowed over at increasing concentrations on the sensor chip in HBS-TE with 1% BSA with 5–8 min association and 10–15 min dissociation. SARS-CoV-2 protein reagents were sourced commercially from Acro Biosystems: S trimer WA1 (SPN-C52H9); S trimer Beta (SPN-C52Hk); S trimer Delta (SPN-C52He); S trimer Kappa (SPN-C52Hr); S trimer Lambda (SPN-C52Hs); S trimer Omicron (SPN-C52Hz); S trimer BA.4 (SPN-C5229); S trimer BA.5 (SPN-C522e); S1 Delta (S1N, C52Hu); S RBD Omicron (SPD-C522e); S RBD BA.2 (SPD-C522g); S RBD BA.3 (SPD-C522i); and S RBD BA.4/5 (SPD-C522r). Following each injection cycle, the surface was regenerated with 2× 30 s injections of IgG elution buffer (Thermo Fisher Scientific). Data were analyzed in Carterra’s Kinetic Tool software with a 1:1 binding model.

Epitope binning was conducted in a premix format using similar biosensor conditions as above. Antibodies were amine-coupled to the HC30M sensor chip by EDC/NHS activation, followed by ethanolamine HCl pH 8.0 quenching. A premixed solution of 66 nM antibody with 5 nM S RBD BA.4/5 (SPD-C522r) was injected over the antibodies coupled to the sensor. Data were analyzed in Carterra’s Epitope Tool software. Competition assignments were determined relative to the binding responses for SARS-CoV-2 S RBD BA.4/5 alone (normalized to 1). Heat maps representing the competition results were generated, where red, yellow, and green cells represent competitive, partially competitive or inconclusive, and non-competitive analyte/ligand pairs, respectively.

ACE2 blocking assays were conducted in a sequential injection format using similar biosensor conditions as above. Antibodies were amine-coupled to the HC30M sensor chip by EDC/NHS activation, followed by ethanolamine HCl pH 8.0 quenching. Experiment was run in HBS-TE with 1% BSA. 10 nM SARS-CoV-2 S RBD BA.4/5 (Acro SPD-C522r) was injected, followed by 10 nM ACE2-Fc (Acro AC2-H5257). Data were analyzed in Carterra’s Kinetics Tool software.

### Alanine scanning mutagenesis

Alanine scanning mutagenesis was performed essentially as described previously [27, 43]. A SARS-CoV-2 (strain Wuhan-Hu-1) S RBD shotgun mutagenesis mutation library was prepared by individually mutating the 184 residues of RBD (335–526) to alanine (or serine if already alanine). Each mutation was confirmed by DNA sequencing, and clones were arrayed in a 384-well plate, one mutant per well. Each S protein mutant was transfected into HEK-293 T cells and allowed to express for 22 hr. Cells were fixed in 4% (v/v) paraformaldehyde (Electron Microscopy Sciences) and permeabilized with 0.1% (w/v) saponin (Sigma-Aldrich) in PBS plus calcium and magnesium (PBS++). Cells were incubated with mAbs diluted in PBS++, 10% normal goat serum (Sigma), and 0.1% saponin. MAb screening concentrations were determined using an independent immunofluorescence titration curve against wild-type S protein to ensure that signals were within the linear range of detection. Antibodies were detected using 3.75 μg/ml of Alexa Fluor488-conjugated secondary antibody (Jackson ImmunoResearch Laboratories) in 10% normal goat serum with 0.1% saponin. Cells were washed three times with PBS++/0.1% saponin followed by two washes in PBS, and mean cellular fluorescence was detected using a high-throughput Intellicyte iQue flow cytometer (Sartorius). Antibody reactivity against each mutant S protein clone was calculated relative to wild-type S protein reactivity by subtracting the signal from mock-transfected controls and normalizing to the signal from wild-type S-transfected controls. Mutations within clones were identified as critical to the mAb epitope if they did not support reactivity of the test mAb but supported reactivity of other SARS-CoV-2 antibodies. This counter-screen strategy facilitates the exclusion of S mutants that are locally misfolded or have an expression defect. Validated critical residues represent amino acids whose side chains make the highest energetic contributions to the MAb-epitope interaction [44, 45].

### Cryo-EM

Lyophilized, his-tagged SARS-CoV-2 S trimer protein was dissolved in 260 μl of MilliQ water. After resting for 30 min at RT, the solution was transferred into a 200 μl dialysis button, covered by a dialysis membrane with a 14 kDa cutoff, and dialyzed at 4°C overnight (17 hr) into PBS, pH 7.4 to remove the trehalose. After confirming protein concentration using a NanoDrop, the solution was used immediately for grid preparation. A thawed RBT-0813 stock was initially diluted 7-fold with PBS, pH 7.4. This dilution was further diluted 4.5- and 6-fold with PBS. 1 μl of the former dilution was mixed with 9 μl S trimer to create a solution with a 3:1 RBT-0813:S trimer ratio; 1 μl of the latter dilution was mixed with 9 μl S trimer to create a solution with a 2:1 RBT-0813:S trimer ratio. Solutions were incubated for 15 min at 4°C and immediately vitrified in liquid ethane.

Movies were acquired on a Titan Krios XFEG, 300 kV, Cs 2.7 mm, Gatan K3 DED (5760 × 4092, 0.83 Å/pixel, 40 frames, 1.1e/Å2/frame) in non-super resolution counting mode. Compensated fringe-free imaging in a 3 × 3 or 5 × 5 beam shift pattern was performed with three expositions per hole using custom SerialEM [46] scripts. Cryo-EM processing was performed using an internally modified RELION [47–50] and Scipion [51] suites. Collected movies were subjected to a motion-search algorithm [52], and both motion-corrected and motion-corrected/dose-weighted micrographs were produced. Motion-corrected micrographs without dose-weighting were used for defocus estimation, while motion-corrected/dose-weighted micrographs were used for further processing.

Particle picking was performed on denoised micrographs using deep learning-based approaches, selecting ~7M potential particles. These potential particles were split into 71 sets of ~100k particles each, and each set was subjected to a “cleaning” 3D classification against a spike-only (i.e. without antibody), low-pass filtered, initial model created earlier in the screening phase of the project, leaving ~1.4M particles showing clear antibody density. These particles were then split into six sets, each with about 233k particles, and each set was further subjected to two rounds of 2D classification (one standard, one suppressing low frequency CTF correction) to create a clean set of 588k particles. A first unmasked consensus refinement was performed on this set, yielding a 3.5 Å map of the spike with strong densities for a VHH bound to a “down” RBD on spike S^A^ (VHH1) and another VHH bound to an “up” RBD on spike S^B^, and a weak density for a third VHH bound to the second “up” RBD on spike S^C^ (VHH3). Following this initial refinement, Bayesian polishing [53] and per-particle defocus refinement improved the resolution to 3.2 Å. This map is called the “initial consensus map” hereafter.

Using the “initial consensus map,” a masked 3D classification of two classes with local searches was performed, where the mask encapsulated the locations of VHH1 and VHH2 and their respective RBDs. This classification separated remaining unbound spike particles (class 1) and particles with strong VHH1 and VHH2 densities (class 2). This class 2, containing 348k particles, was then used for a masked 3D refinement with local searches, producing the 3.4 Å map used to build the majority of the VHH1 epitope/paratope. Since the density for VHH2 was still suboptimal, additional masked 3D refinement with local searches was performed, but with a mask specifically only around VHH2 and its corresponding RBD-up location. This refinement produced the 3.3 Å map used to build the VHH2 epitope/paratope.

VHH3 was clearly visible in the “initial consensus map” but too weak to interpret. Thus, the “initial consensus map” was used as a basis for no-align 3D classification into six classes. This 3D classification revealed four classes that represented either unbound, all RBD down spike or spike with a very weak density of VHH 3 and 2 classes with a stronger density of VHH3. These two classes, comprising 274k particles, were then combined and subjected to an unmasked 3D refinement that yielded the 3.3 Å map referred to as the “global consensus map.” Upon convergence, however, the density for the VHH3 was already misaligned due to the presence of the spike protein. Indeed, 3.3 Å represents the resolution of the spike protein, not the true resolution of the VHH3 part of the map. Most reliable fitting of the VHH3 density could be done using map from iteration 8 of this global consensus refinement, which yielded the 6 Å map used to assign the position of VHH3. Further attempts at improving the density of VHH3 using similar approaches as those used for VHH1 and VHH2 did not bring any improvement. The most likely reason is that while VHH1 and VHH2 are rigidly bound to their respective RBD domains, VHH3 appears to be only flexibly bound to its RBD domain, and the mass of VHH3 itself is too small to refine properly on its own.

Finally, the “global consensus map” was used as a basis for multi-body refinement [54] that encapsulated VHH1 and parts of its surrounding RBD domain and especially the N-term domain of the neighboring B chain as one body (with spike core being the second body). This multi-body refinement yielded the 3.7 Å map that resolved the N-term interface well and was used to build the N-term B chain epitope of VHH1.

Initially, pdb:6x2b was used to rigid-body fit the map densities. Afterwards, all relevant residues were manually remodeled to correspond to the map density. The sequence of 6x2b was corrected to include all the amino acids present in the spike construct, which also aligned it such that the amino acid numbers correspond to the mutagenesis numbering. The model building then proceeded iteratively, combining restrained molecular dynamics with manual intervention using Coot [55] to build stereochemically valid models with best possible correspondence to the density.

A similar approach was adopted for building the VHH models, only here AlphaFold2 [56] predictions of the N-term and C-term VHH domains were used as a starting point for the rigid body fitting and subsequent manual/molecular dynamics remodeling.

### Pseudovirus neutralization assay (Figs 3a and 7a)

Three-fold serial dilutions of antibody were prepared in DMEM with 2% FBS and incubated with rVSV-SARS-CoV-2 (Wuhan) or rVSV-SARS-CoV-2 (Omicron BA.1) for 1 hr at RT. Media was removed from Vero cells in a 96-well plate, and 65 μl of virus/antibody mixture was added to each well in triplicate. The cells were incubated at 37°C and 5% CO_2_ for 10 hr. The cells were then fixed with 4% paraformaldehyde, washed with 1x PBS, and stored in 1x PBS with Hoechst 33342 (Invitrogen) at a dilution of 1:2000. Viral infection was quantified by automatic enumeration of GFP-positive cells from captured images using Cytation 5 automated fluorescence microscope (BioTek) with analysis done by Gen5 data analysis software (BioTek). Data analysis was conducted using Prism software (GraphPad).

### RBT-0813, TB618-065, and TB493-011 pseudovirus neutralization assay (Figs 3d, 5a and b, and 7b and c)

The pseudovirus neutralization assay was performed as described previously [27]. Serial semi-log dilutions of antibody were prepared and mixed with the VSV-pseudotyped with SARS-CoV-2 S in a 1:1 ratio for 1 hr at RT, followed by incubation over Vero cells (ATCC® CCL-81TM) seeded at 60 000 cells per well at 37°C + 5% CO_2_. The cells were lysed the following day, and luciferase activity was measured to assess the potency of each antibody to block viral entry into the Vero cells. All samples were tested in triplicate. Data analysis was conducted using XLFit and Prism software (GraphPad).

### ELISA

ELISA was conducted using Nunc 96-well Maxisorp plates (Thermo) with passively absorbed SARS-CoV-2 S RBD Omicron BA.1.1.529 (Acro SPD-C522e) or SARS-CoV-2 S RBD BA.4/5 (Acro SPD-C522r). MonoRab Rabbit Anti-Camelid VHH Antibody [HRP] (Genscript) was used to detect the presence of bound antibodies following the addition of 3,3′,5,5′-TMB substrate.

### Escape assays

Antibody escape mutants were isolated as described [57]. Briefly, Vero cells (ATCC) were cultured in DMEM supplemented with 2% heat-inactivated fetal bovine serum (FBS; Bio-Techne), 1% penicillin–streptomycin (ThermoFisher Scientific), and 1% GlutaMAX (ThermoFisher Scientific). Cells were plated in 6-well plates (Corning) and antibody concentrations that afforded 90% viral neutralization (IC_90_) were estimated from baseline neutralization curves of rVSV-SARS-CoV-2 (Wuhan). Five-fold serial dilutions of rVSV-SARS-CoV2 (Wuhan) were prepared and incubated with antibodies for 1 hr at RT. Media was then removed, and virus/antibody mixture was added. Cells were incubated at 37°C and 5% CO_2_ and monitored daily for evidence of viral eGFP reporter expression by fluorescence microscopy. Once infection and spread were observed, virus-containing supernatants were harvested and used for the next serial passage. Antibody concentrations were doubled in every passage. Following evidence of neutralization resistance, viral clones were plaque-purified from supernatants and amplified, as described [57]. The virus-encoded spike gene was amplified by reverse-transcription PCR as described and subjected to Sanger sequencing.

### Authentic virus neutralization assay

Antibodies were serially 1:3 diluted with independent triplicates in sterile DPBS (Sigma) to a range of 12, 400–0.2 ng/ml. Negative control wells (*n* = 8 wells per 96-well plate) contained sterile DPBS with no monoclonal antibody. Diluted antibodies were mixed with an equal volume of SARS-CoV-2 BA.5 Omicron, strain COR-22-063113/2022 (World Reference Center for Emerging Viruses and Arboviruses, UTMB, Galveston, TX), diluted to an initial concentration of 5000 focus-forming units (FFU)/ml in sterile DPBS. The virus:antibody mixture was allowed to incubate for 1 hr at 37°C with 5% CO_2_. Then, 20 μl of the virus:antibody mixture was allowed to infect a confluent monolayer of Vero TMPRSS2 cells (JCRB1819, JCRB Cell Bank, Ibaraki, Japan) in a 96-well plate for 1 hr at 37°C with 5% CO_2_. Following infection, cells were overlaid with a solution of 85% MEM (Gibco) and 15% DMEM (Gibco) supplemented with 1 mg/ml geneticin (Sigma), 2% heat-inactivated filtered FBS (R&D Systems), and 0.85% methyl cellulose (Sigma). After two days, the monolayers were fixed with formalin (Fisher) for at least 24 hr. Monolayers were washed with DPBS and incubated in permeabilization buffer consisting of DPBS supplemented with 0.1% BSA (Sigma) and 0.1% saponin (Sigma) for 30 min at RT. Permeabilization buffer was removed, and monolayers were incubated overnight at 4°C with rabbit polyclonal antibody against SARS-CoV N protein (courtesy of Dr. Shinji Makino, Department of Microbiology and Immunology, UTMB, Galveston, TX) diluted in permeabilization buffer. Excess antibody was washed away with DPBS, and monolayers were incubated for 1 hr at RT with HRP-conjugated goat anti-rabbit IgG (cell signaling) diluted in permeabilization buffer. Excess antibody was washed away with DPBS, and foci were stained using KPL TrueBlue Peroxidase Substrate (SeraCare). Once foci were visible under a light microscope, excess substrate was removed, and the monolayers were washed with water. Wells were imaged using the Cytation7 Imagining Reader (BioTek). Foci were counted manually. Percent neutralization was calculated using the mean value of the negative control wells within a given 96-well plate as the baseline (range: 52–56 FFU). Antibody concentrations were log_10_ transformed, and curves were fit using the log(agonist) versus response—Find ECanything function of Graphpad Prism 9.4.0 (GraphPad Software).

## Supplementary Material

Yang_et_al_Supplementary_final_clean_3_12_24_tbae009

## Data Availability

The authors confirm that the data supporting the findings of this study are available within the article and its Supplementary Materials, or upon reasonable request to the corresponding author.
