# Supplementary material for: A VHH single-domain platform enabling discovery and development of monospecific antibodies and modular neutralizing bispecifics against SARS-CoV-2 variants
Source: Antib Ther. 2024 May 3;7(2):164–76. doi: 10.1093/abt/tbae009 (PMC11200683; doi:10.1093/abt/tbae009)
Supplement: Yang_et_al_Supplementary_final_clean_3_12_24_tbae009 [file yang_et_al_supplementary_final_clean_3_12_24_tbae009.docx]

**Supplementary**

**
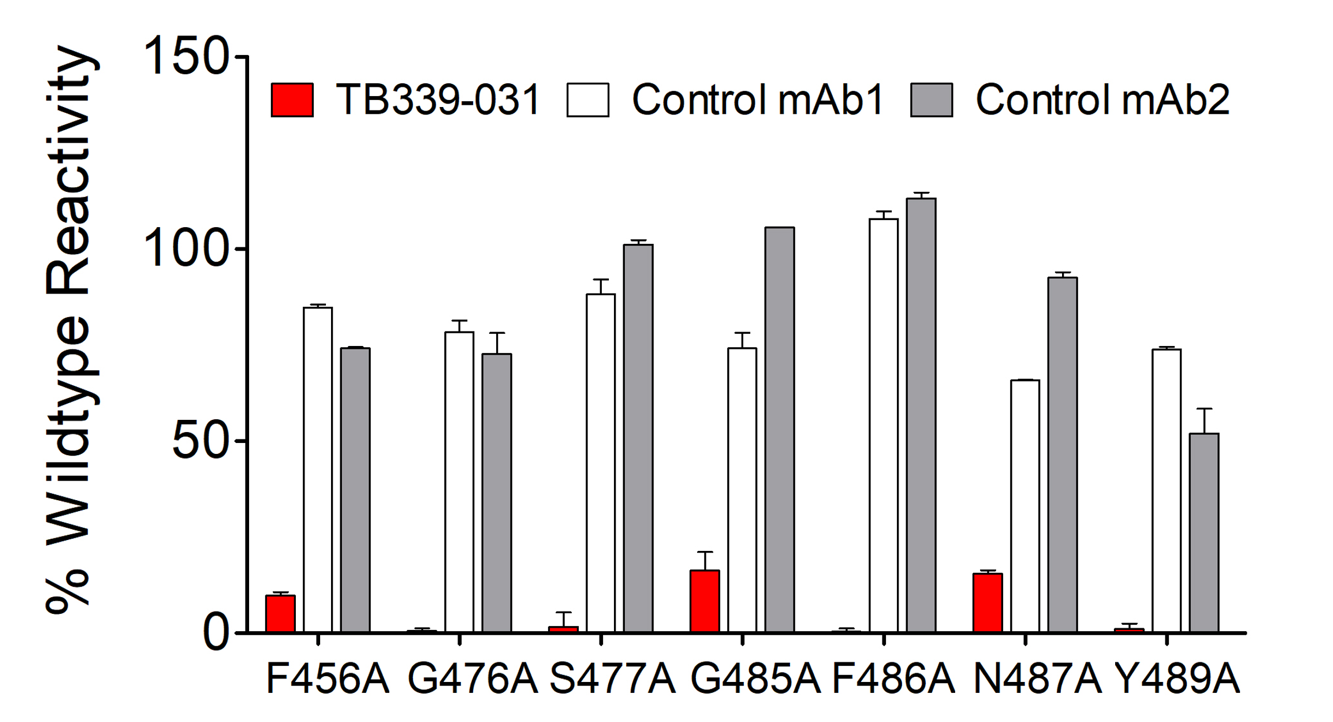
**

**Supplementary Figure 1**

**
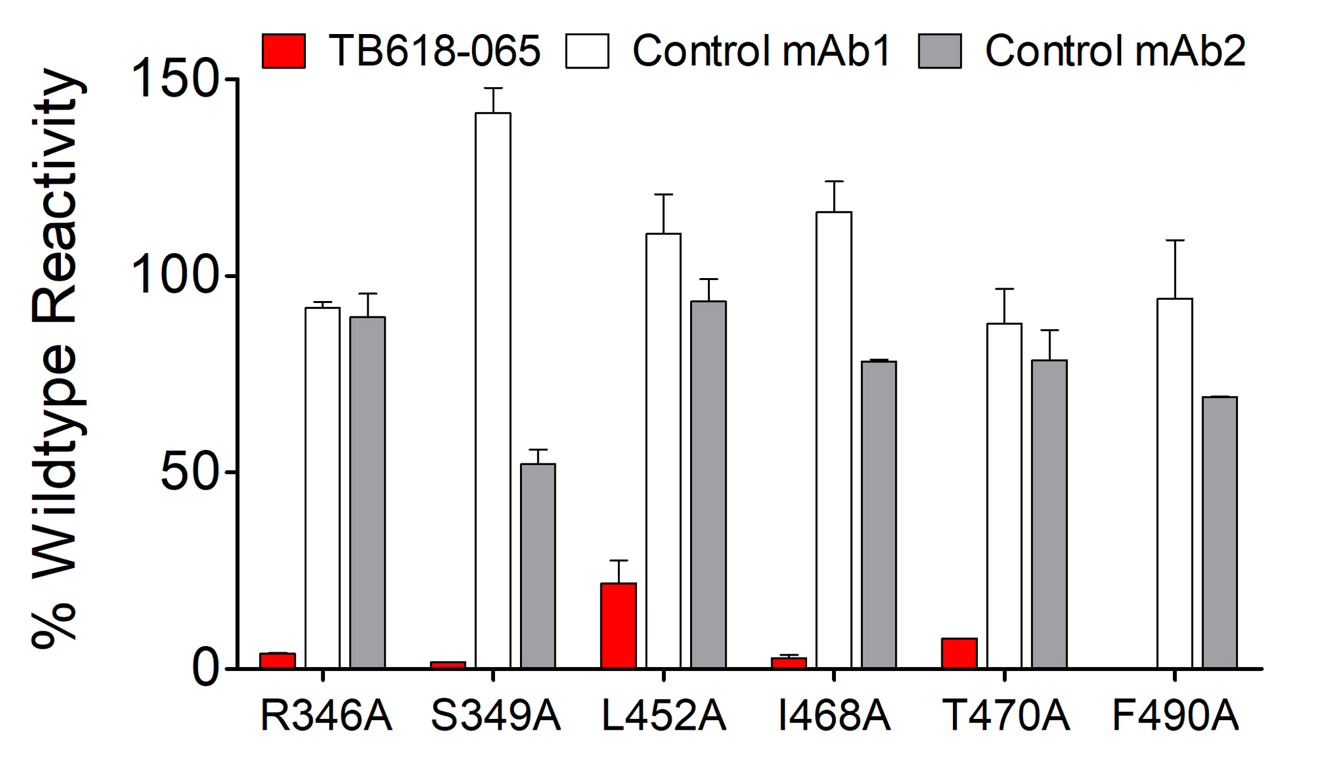
**

**Supplementary Figure 2. Graph shows shotgun mutagenesis epitope mapping binding data of TB618-065.** MAbs were epitope mapped by screening on a SARS-CoV-2 S protein RBD alanine scan mutation library expressed in HEK 293T cells, with binding assayed by flow cytometry. For RBD residues R346, S349, L452, I468, T470, and F490, mutation to alanine greatly reduced TB618-065 binding (red bars) but did not affect binding of two control anti-RBD MAbs (white and light gray bars). Binding values are shown as a percentage of the antibodies’ binding to wildtype SARS-CoV-2 S protein, and represent the mean of at least two replicate data points, with error bars showing half of the range (half of the maximum minus minimum value).

**Supplementary Table 1.** Potential interacting amino acid residues in the TB202-3 epitopes on S^A^ and S^B^, as identified by PDBe PISA (55) based on the coordinates built into the cryo-EM map. Interacting amino acids were determined via two complementary strategies—1) manually inspecting neighboring spike/VHH residues during model building, and 2) leveraging computational methods that analyze residue interfacing based on solvent-accessible area, buried surface area, and solvation area. Residues that were identified in the TB202-3 epitopes in both cryo-EM and also alanine scanning mutagenesis assay are depicted in bold.

| **Epitope 1 (VHH1)** | | **Epitope 2 (VHH2)** |
| --- | --- | --- |
| **S^A^** | **S^B^** | **S^B^** |
| R346 | T108 | A344 |
| F347 | T109 | R346 |
| A348 | S112 | F347 |
| S349 | K113 | A348 |
| Y351 | Q115 | S349 |
| A352 | E132 | Y351 |
| W353 | N164 | A352 |
| N354 | C166 | N354 |
| R355 | T167 | R355 |
| K356 | I233 | K356 |
| K444 |  | S399 |
| Y449 |  | Y449 |
| **N450** |  | **N450** |
| L452 |  | L452 |
| R466 |  | R466 |
| I468 |  | I468 |
| S469 |  | S469 |
| T470 |  | T470 |
| E471 |  | E471 |
| P479 |  | **I472** |
| C480 |  | P479 |
| N481 |  | C480 |
| G482 |  | N481 |
| V483 |  | **F490** |
| C488 |  | L492 |
| **F490** |  | S494 |
| L492 |  |  |
| S494 |  |  |


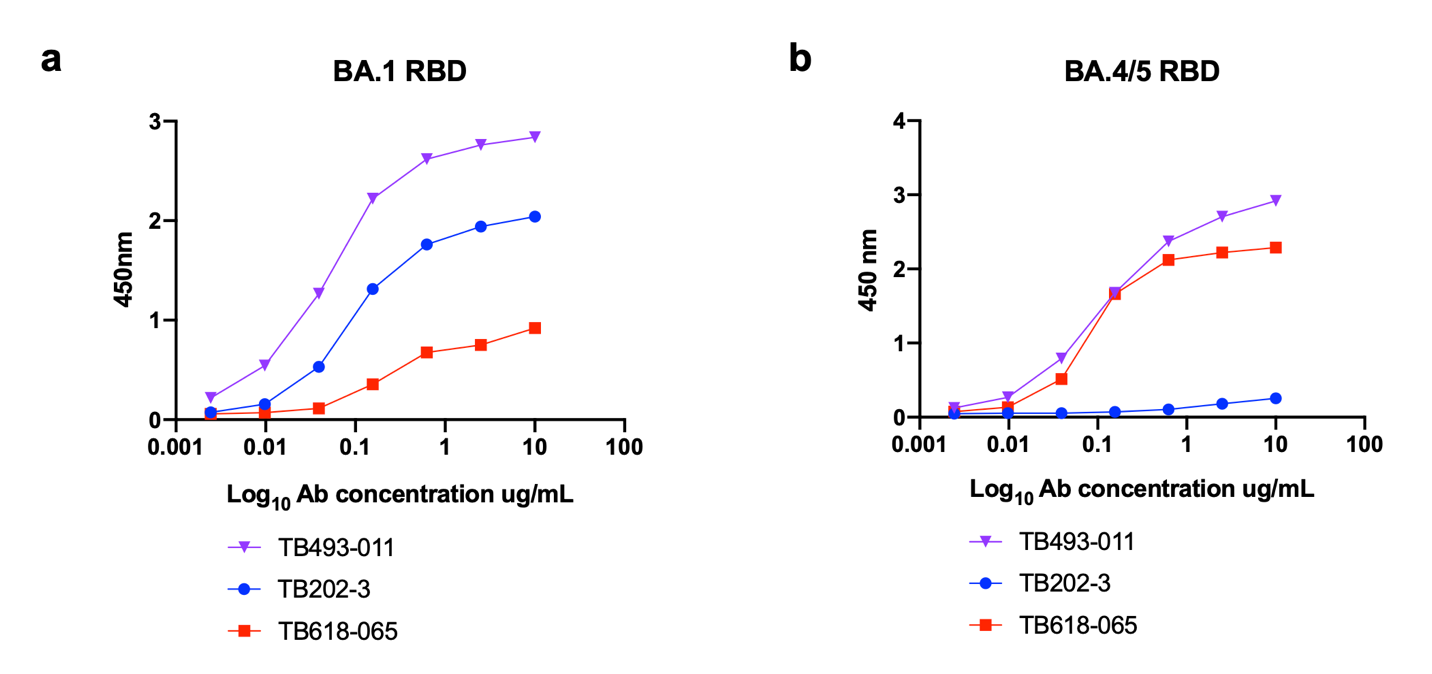


**Supplementary Figure 3.** (A-B) TB493-011, TB202-3, and TB618-065 were tested via ELISA to evaluate binding activity against recombinant BA.1 and BA.4/5 receptor binding domain.


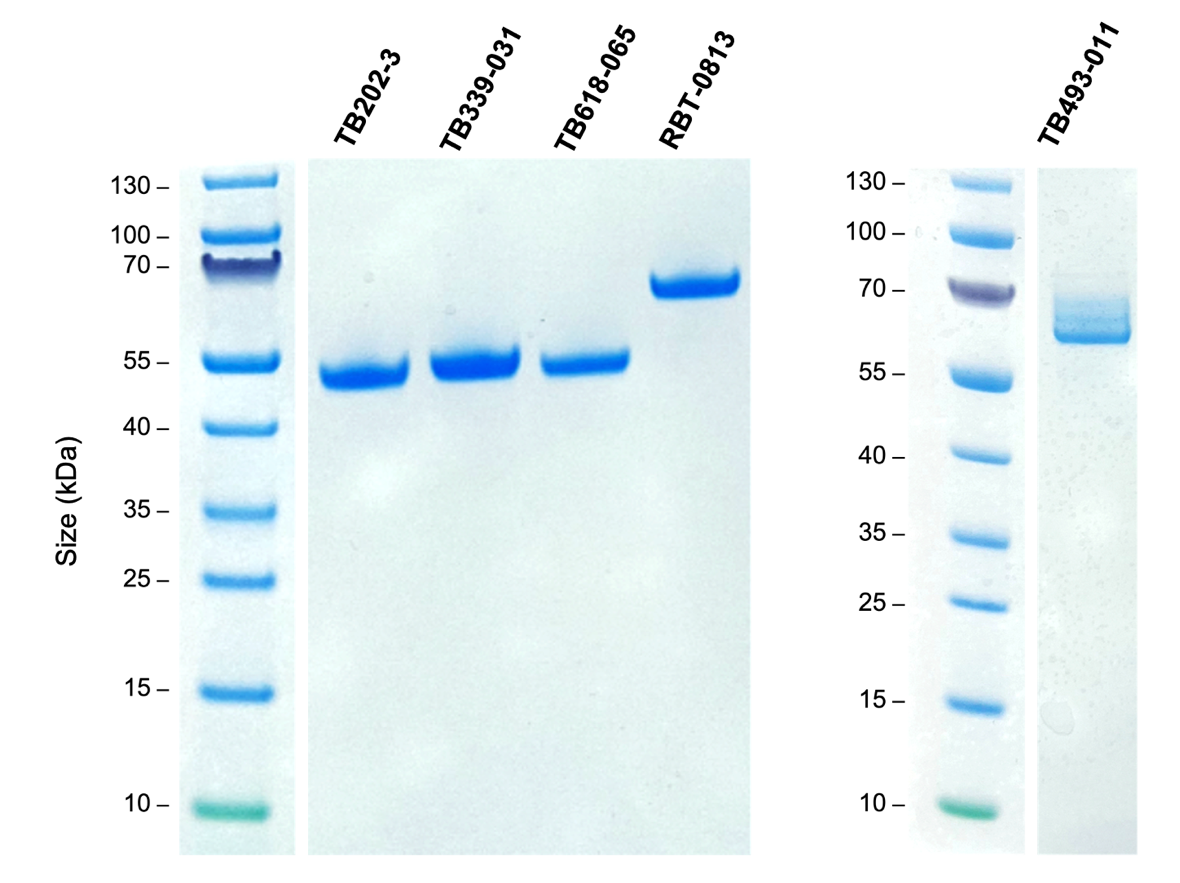


**Supplementary Figure 4.** Sodium-Dodecyl Sulfate-Polyacrylamide Gel Electrophoresis (SDS-PAGE) assay of TB202-3, TB339-031, TB618-065 VHH-Fc constructs, and RBT-0813 & TB493-011 tetravalent VHH-Fc-VHH constructs.
